# Using ex vivo radioactivity measurement to differentiate parathyroid single-gland and multigland disease

**DOI:** 10.1093/oncolo/oyaf091

**Published:** 2025-05-16

**Authors:** Rongzhi Wang, Zhixing Song, Claren Harper, Polina V Zmijewski, M Chandler McLeod, Andrea Gillis, Brenessa Lindeman, Herbert Chen, Jessica Fazendin

**Affiliations:** Department of Surgery, Heersink School of Medicine, University of Alabama at Birmingham, Birmingham, AL 35294-0012, United States; Department of Surgery, Heersink School of Medicine, University of Alabama at Birmingham, Birmingham, AL 35294-0012, United States; Department of Surgery, Heersink School of Medicine, University of Alabama at Birmingham, Birmingham, AL 35294-0012, United States; Department of Surgery, Heersink School of Medicine, University of Alabama at Birmingham, Birmingham, AL 35294-0012, United States; Department of Surgery, Heersink School of Medicine, University of Alabama at Birmingham, Birmingham, AL 35294-0012, United States; Department of Surgery, Heersink School of Medicine, University of Alabama at Birmingham, Birmingham, AL 35294-0012, United States; Department of Surgery, Heersink School of Medicine, University of Alabama at Birmingham, Birmingham, AL 35294-0012, United States; Department of Surgery, Heersink School of Medicine, University of Alabama at Birmingham, Birmingham, AL 35294-0012, United States; Department of Surgery, University of Oklahoma, 825 NE 10th Street, Oklahoma City, OK 73104, United States

**Keywords:** parathyroidectomy, radioguided, ex vivo, radioactivity

## Abstract

**Background:**

Radioguided parathyroidectomy is based on the principle that hyperfunctioning parathyroid glands have increased radiotracer uptake, which can be measured instantaneously. We sought to determine if ex vivo radioactivity measurement could be used to predict parathyroid pathology and guide surgical decision-making in a time-efficient manner.

**Materials and Methods:** We retrospectively reviewed patients with primary hyperparathyroidism who underwent parathyroidectomy at our institution (2000-2022). All patients received a preoperative injection of 10mCi of ^99^Tc. Intraoperatively, radioactivity was measured by a gamma counter within 5 seconds. The ratio of radioactive counts of resected parathyroid glands (ex vivo) to background thyroid tissue (radioactive ratio [RR]) was calculated. Patient demographics, preoperative laboratory measurements, and RRs were compared between patients with single-gland disease (SGD) versus multigland disease (MGD). The predictive threshold for SGD was subsequently validated on the following cohort of 115 patients.

**Results:**

Of 2368 patients included, 1585 (66.9%) patients had SGD, and 783 (33.1%) had MGD. Patients with SGD had higher median (IQR) RRs than the MGD group (0.8 [0.5-1.3] vs 0.4 [0.3-0.7], *P* <.001). After adjusting for age, preoperative calcium and PTH, reoperative parathyroidectomy, and gland weight, the RR was an independent predictor (OR 2.155, *P* <.001) of SGD. A receiver operating characteristic curve was plotted using the RR to predict the likelihood of SGD. The positive predictive value (PPV) reached a plateau at 85.3% when RR was 1.2. When the threshold of 1.2 was used in the validation cohort, the PPV was 92.3%.

**Conclusions:**

Ex vivo radioactivity measurement provides an instantaneous and reliable prediction of parathyroid pathology, which could be used as an adjunct to guide surgical decision-making during parathyroidectomy.

**Implications for Practice**

The findings of this study suggest that intraoperative radioactivity measurement can be a valuable tool for parathyroid surgeons in distinguishing between single-gland disease (SGD) and multigland disease. The threshold of a radioactivity ratio ≥1.2 demonstrated a high positive predictive value (85.3%) for SGD, offering a reliable and time-efficient method to guide surgical decisions. Integrating this technique with intraoperative parathyroid hormone monitoring may reduce the need for more invasive or prolonged procedures, potentially improving patient outcomes. Surgeons could use this simple and rapid intraoperative tool to help determine whether a bilateral neck exploration is necessary, ultimately optimizing surgical planning and minimizing unnecessary wait times in the operating room.

## Introduction

Primary hyperparathyroidism (HPT) is a common endocrine disorder caused by the autonomous overproduction of parathyroid hormone (PTH) from hyperfunctioning parathyroid glands. Most patients with primary hyperparathyroidism (~60%-80%) have a single adenoma, while the remaining have more than one hyperfunctioning parathyroid gland. Untreated HPT can lead to end organ dysfunction such as kidney stones, abnormal renal function, reduced bone density, and neuropsychiatric symptoms. Parathyroidectomy is the only definitive treatment of primary HPT, effectively normalizing biochemical profiles, alleviating symptoms, and improving quality of life.^1,2^ With the advancement of imaging modalities and intraoperative adjuncts, minimally invasive parathyroidectomy (MIP) has been widely adopted to minimize exploration, enhance recovery, and reduce postoperative complications.^3,4^

Preoperative imaging such as cervical ultrasonography (US), technetium-labeled sestamibi scintigraphy (sestamibi scan), and 4-dimensional computed tomography (4D CT) is frequently used for localization. However, these radiologic studies could be inaccurate in localizing disease or distinguishing between single-gland disease (SGD) and multigland disease (MGD).^4^ A previous study from our group on preoperative parathyroid localization found that, although 4D CT was the most sensitive study (92%) in detecting abnormal parathyroid glands, the specificity of detecting all abnormal parathyroid glands was only 64%.^5^ MGD typically presents with smaller lesions that are often difficult to identify on imaging. Based on the high negative findings of 4D CT scans for MDG, Sephahdari et al. developed a 4D CT MGD score based on the number and size of lesions.^6,7^ The highest score is assigned to cases with multiple lesions, no lesions, or lesions smaller than 7 mm. The MGD scores of ≥2, ≥3, and 4 had specificites of 68%, 81%, and 96%, respectively. While the scoring system demonstrates high specificity, it fails to fulfill the original purpose of providing the anatomical detail necessary for surgical planning.

Intraoperative PTH (IoPTH) monitoring is commonly used as an adjunct during parathyroidectomy to determine the extent of exploration and the success of the operation. It provides a real-time assessment of parathyroid function and a high cure rate (97%-99%) when used in conjunction with MIP.^4^ The time to result for commercially available assays used for IoPTH ranges from 12 to 20 minutes.^8^ Nevertheless, there are practical challenges that can extend the testing turnaround time, such as the availability of testing assays and instruments, transportation of samples from the operating room (OR), and delays in communicating results. The testing turnaround time can vary from 20 to more than 40 minutes.^9^ If surgeons remove an abnormal parathyroid gland from one side and then wait for IoPTH result, there is a chance that the IoPTH level will not drop appropriately due to MGD. In such cases, surgeons would have to convert to bilateral neck exploration after a potentially avoidable wait time. A prolonged wait time for IoPTH or unnecessary delays may result in higher operating costs and increased intraoperative risks associated with general anesthesia.

Radioguided parathyroidectomy is a frequently used surgical technique during MIP. It involves preoperative intravenous injection of technetium^99m^ (^99^Tc) and measuring radioactivity of the thyroid gland (background count) and resected parathyroid gland (ex vivo count) intraoperatively.^10^ The principle of radioguided parathyroidectomy is that ^99^Tc attaches to the mitochondria, which are abundant in hyperfunctioning parathyroid glands. Any excised tissue during parathyroidectomy containing more than 20% of background radioactivity indicates hyperfunctioning parathyroid tissue.^10,11^ Radioguided parathyroidectomy offers an instantaneous measurement of radioactivity, excellent cure rates for primary HPT, eliminates intraoperative frozen section, and reduces operative time.^12^ In a previous study, it was observed that the ex vivo count was higher in patients with SGD compared with MGD.^10^ It is currently unknown whether there is a specific threshold ex vivo count that can differentiate between SGD and MGD. Therefore, we aim to investigate whether ex vivo activity measurement could be utilized to predict parathyroid pathology and guide surgical decision-making during parathyroidectomy.

## Methods

We retrospectively reviewed patients with primary HPT who underwent radioguided parathyroidectomy at our institution from 2000 to 2022. Patients with persistent HPT after parathyroidectomy were excluded. In the preoperative holding area, patients were administered 10mCi of ^99^Tc sestamibi 30-60 minutes prior to the operation. Radioactivity was measured by a gamma counter, which provided data within 5 seconds. In the OR, we use a handheld, wireless gamma probe, Neoprobe (Devicor Medical Products, Inc. 2021. Cincinnati, OH), to detect tissue radioactivity.^13^ Before exploring the parathyroid, the gamma probe is positioned on the thyroid isthmus to establish a background count for radioactivity. After removing an abnormal parathyroid gland, it is placed in the center of a gamma probe. The measurement of radioactivity count from the removed gland is termed “ex vivo count.” The radioactivity ratio (RR) is calculated as the ratio of the ex vivo count to the background count. A resected parathyroid gland with RR ≥ 0.2 is considered abnormal and hypercellular. Only the ex vivo count and RR of the largest gland were reported in patients with MGD. Intraoperative PTH (IoPTH) monitoring is utilized in all cases. The baseline PTH levels were collected in the preoperative holding area. PTH levels were checked at 5-, 10-, and 15-minutes after resection of all abnormal parathyroid glands. The endpoint of the operation was an IoPTH drop of ≥50%.

SGD is defined as the identification of only one hypercellular parathyroid gland on the final pathology report. Multigland disease is defined as the identification of more than one hypercellular parathyroid gland. Patient demographics, preoperative laboratory results, parathyroid gland weight, and radioactivity measurements were compared between patients with SGD versus MGD.

Descriptive statistics were performed to describe the characteristics of each group. Means and standard deviations (SD) were used for continuous variables with normal distribution, while nonnormal variables were described using medians and interquartile ranges (IQRs). The categorical variables were summarized using counts and percentages. Bivariate analyses were conducted using independent *t*-tests, Mann-Whitney U, and χ2 tests. Binary logistic regression was performed to evaluate the predicting factors for SGD. The receiver operating characteristic (ROC) curve analysis was performed to identify the optimal cutoff value of RR to predict SGD. The sensitivity and specificity of each coordinate of the ROC curve were obtained. Positive predictive value (PPV) and negative predictive value (NPV) were calculated as follows: PPV = (sensitivity * prevalence)/[(sensitivity*prevalence) + ((1-specificity) * (1-prevalence))] and NPV = (specificity * (1-prevalence))/[(specificity * (1- prevalence)) + ((1-sensitivity) * prevalence)]. The best cutoff value was determined by the PPV. The predictive threshold for SGD was subsequently validated on the following cohort of 115 patients. IBM SPSS version 29.0 was used for data analysis. Variables were considered statistically significant at *P* <.05. This study was approved by the UAB Institutional Review Board.

## Results

Of 2368 patients included, 1585 (66.9%) patients with SGD and 783 (33.1%) had MGD. Most patients were female (78.3%) and White (92.2%), with a mean age of 60 ± 14 years (Table 1). Patients with SGD were younger than those with MGD (60 ± 14 vs 58 ± 15, *P* =.001). There was no difference in sex, race/ethnicity, or body mass index (BMI) between the 2 groups. There were more patients in the SGD group with a history of previous parathyroidectomy than in the MGD group (7.6% vs 12.0%, *P* <.001). Compared with patients with MGD, patients with SGD had higher preoperative calcium (11.0 ± 0.8 vs 10.5 ± 0.8 mg/dL, *P* <.001) and higher preoperative PTH (109 [83-151] vs 89 [65-125] pg/mL, *P* <.001).

**Table 1.** Patient demographic and preoperative laboratory results.

	SGD (*n* = 1585)	MGD (*n* = 783)	Total (*n* = 2368)	*P*-value
Age	60 ± 14	58 ± 15	60 ± 14	.001
Sex				.159
Female	1227 (77.4%)	626 (79.9%)	1853 (78.3%)	
Male	358 (22.6%)	157 (20.1%)	515 (21.7%)	
Race				.575
White	1458 (92.3%)	728 (91.9%)	2176 (92.2%)	
Black/African American	82 (5.2%)	43 (5.5%)	125 (5.3%)	
Asian	30 (1.9%)	18 (2.3%)	48 (2.0%)	
Hispanic	10 (0.6%)	2 (0.3%)	12 (0.5%)	
Missing data	5 (0.3%)	2 (0.3%)	7 (0.3%)	
BMI, kg/m^2^, mean ± SD	30.9 ± 7.4	30.7 ± 8.2	30.9 ± 7.7	.475
Reoperation	121 (7.6%)	94 (12.0%)	215 (9.1%)	<.001
Preop calcium, mg/dL, mean ± SD	11.0 ± 0.8	10.5 ± 0.8	10.9 ± 0.8	<.001
Preop PTH, pg/mL, median (IQR)	109 (83-151)	89 (65-125)	102 (77-143)	<.001
Preop Cr, mg/dL, mean ± SD	0.96 ± 0.31	0.94 ± 0.30	0.95 ± 0.31	.293

Abbreviations: BMI, body mass index; Cr, creatinine; MGD, multigland disease; Preop, preoperative; PTH, parathyroid hormone; SD, standard deviation; SGD, single-gland disease.

The background counts were similar between the 2 groups. However, compared with the MGD group, the SGD group had a higher ex vivo count (163 [92-290] vs 95 [52-161], *P* <.001) and a higher RR (0.8 [0.5-1.3] vs 0.4 [0.3-0.7], *P* <.001) (Table 2).

**Table 2.** Intraoperative findings.

	SGD (*n* = 1585)	MGD (*n* = 783)	Total (*n* = 2368)	*P*-value
Background count	199 (142-274)	207 (145-285)	200 (142-278)	.133
Ex vivo count	163 (92-290)	95 (52-161)	134 (73-246)	<.001
RR	0.8 (0.5-1.3)	0.4 (0.3-0.7)	0.7 (0.4-1.2)	<.001
Largest gland weight	452 (241-869)	311 (178-675)	413 (220-800)	<.001

Abbreviations: MGD, multigland disease; RR, radioactivity ratio; SGD, single-gland disease.

**Figure 1.**
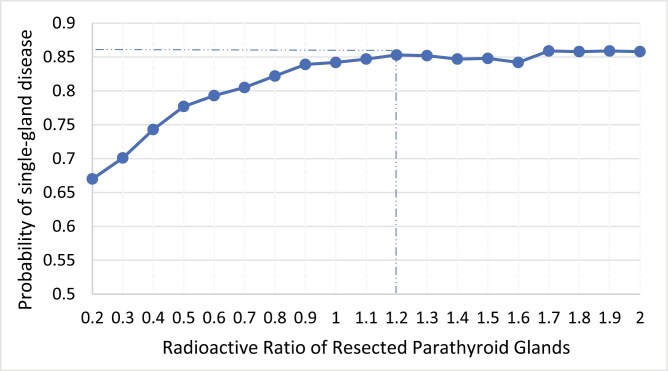
Probability of single-gland disease in relation to radioactive ratio.

In the binary logistic regression model, older age, higher preoperative calcium and PTH, no previous history of parathyroidectomy, and higher RR were independently associated with SGD (Table 3). The adjusted odds ratio (OR) of RR was 2.155 (95% CI, 1.791-2.593, *P* <.001). The area under the curve of the ROC curve was 0.715. The coordinates of the ROC curve are shown in Table 3. When the RR was greater than or equal to 1.2, the PPV plateaued at 85.3% (Figure 1). Although the PPVs were slightly higher than 85.3% when RR > 1.7, we did not choose them as optimal cutoff values because the incidences dropped below 10.6% (Table 4). When examined using the cutoff value of 1.2 in the validation cohort, the PPV was 92.3%, while the sensitivity, specificity, and NPV were 53.7%, 93.7%, and 59.2%, respectively.

**Table 3.** Binary logistic regression to predict SGD.

Variable	OR	95% CI	*P*-value
Age	1.010	1.003-1.016	.004
Preoperative calcium	2.267	1.966-2.614	<.001
Preoperative PTH	1.000	1.000-1.001	.335
Previous history of PTx	0.687	0.492-0.904	.009
RR	2.155	1.791-2.593	<.001
Largest gland weight	1.000	1.000-1.000	.112

Abbreviations: OR, odds ratio; PTx, parathyroidectomy; RR, radioactivity ratio; SGD, single-gland disease.

**Table 4.** Test outcomes of predicting the probability of single-gland disease in relation to radioactive ratio.

RR	Accumulative %	Sensitivity (TP/TP + FN)	Specificity (TN/TN + FP)	PPV (TP/TP + FP)	NPV (TN/TN + FN)
0.2	9.2%	99.2%	1.5%	67.0%	50.0%
0.3	20.5%	94.3%	18.5%	70.1%	61.7%
0.4	30.2%	87.7%	61.2%	74.3%	61.0%
0.5	39.4%	80.50%	53.40%	77.70%	57.50%
0.6	47.0%	71.40%	62.30%	79.30%	51.80%
0.7	54.6%	63.20%	69.10%	80.50%	48.10%
0.8	61.0%	55.00%	75.90%	82.20%	45.50%
0.9	65.7%	48.30%	81.20%	83.90%	43.70%
1	69.0%	42.90%	83.80%	84.20%	42.10%
1.1	74.0%	37.80%	86.20%	84.70%	40.70%
1.2	77.4%	32.90%	88.50%	85.30%	39.50%
1.3	80.5%	28.80%	89.90%	85.20%	38.40%
1.4	83.2%	24.60%	91.10%	84.70%	37.40%
1.5	85.7%	21.20%	92.30%	84.80%	36.70%
1.6	87.6%	17.90%	93.20%	84.20%	36.00%
1.7	89.4%	15.80%	94.80%	85.90%	35.70%
1.8	90.7%	13.30%	95.50%	85.80%	35.30%
1.9	91.6%	11.90%	96.00%	85.90%	35.00%
2	92.7%	10.70%	96.40%	85.80%	34.80%

Abbreviations: FN, false negative; FP, false positive; NPV, negative predictive value; PPV, positive predictive value; RR, radioactivity ratio; TP, true positive.

## Discussion

The decision to perform an MIP and bilateral neck exploration remains challenging for parathyroid surgeons because there are no perfect predictors to differentiate SGD and MGD. Our study discovered that older age, higher preoperative calcium and PTH, absence of previous parathyroidectomy, and a higher radioactivity ratio of the resected parathyroid glands were independently associated with SGD. The threshold of RR greater than or equal to 1.2 can accurately predict SGD etiology with a PPV of 85.3%. Besides, the intraoperative measurement of radioactivity is immediate, within 5 seconds. This suggests that radioactivity measurement can be utilized to predict parathyroid pathology in a time-efficient manner.

Radioguided parathyroidectomy was first described by Norman et al. in 1997.^14^ Norman et al. proposed a “20% rule” to identify parathyroid adenomas: a resected parathyroid gland with more than 20% RR and a positive sestamibi scan for parathyroid adenomas.^11^ Further studies by Chen et al. demonstrated that “20% rule” for ex vivo counts applied not only to parathyroid adenomas but also to hyperplastic glands in patients with primary HPT.^10,15^ The radioguided parathyroidectomy was originally performed only on patients with a positive sestamibi scan for parathyroid adenomas. It was thought that a negative sestamibi scan indicated that the abnormal parathyroid glands would not absorb ^99^Tc and, therefore, would not be detected using a gamma probe during surgical exploration. Chen et al. conducted a study of 769 patients with primary HPT who had a preoperative sestamibi scan.^16^ Among them, 134 had a negative sestamibi scan. The study found that patients with negative sestamibi scans had a lower mean of in vivo counts (258 vs 301, *P* <.01) and ex vivo counts (47 vs 90, *P* <.01) compared with those with positive sestamibi scans. Nevertheless, all abnormal parathyroid glands in both groups exhibited ex vivo counts higher than 20% of background counts, indicating that radioguided techniques are equally effective in patients with negative sestamibi scans for primary HPT. One concern regarding radioguided parathyroidectomy was the potential radiation exposure to surgeons and other OR staff. Oltmann et al. conducted a study over 6 months, measuring radiation exposure by having surgeons and OR staff wear dosimetry badges in 68 cases.^17^ The study concluded that the radiation exposure from radioguided parathyroidectomy was minimal. Surgeons had a mean deep dose exposure of 0.8 ± 0.2 mrem/case. The scrub tech’s exposure was 1/2-2/3 that of the surgeons, and anesthesia exposure was minimal. According to their institutional protocol, only surgeons performing more than 625 radioguided parathyroidectomy per year require routine radiation exposure monitoring. Nowadays, radioguided parathyroidectomy has been performed on a wider range of patients, including those with secondary and tertiary HPT,^18^ Familial HPT,^19^ and reoperative neck.^20^ Multiple studies have shown that radioguided parathyroidectomy offers the benefits of reducing surgical timing, eliminating intraoperative frozen sections, and locating ectopic parathyroid glands during the operation, with an excellent cure rate.^10,12,21^ In cases with missing parathyroid glands or insufficient drop in IoPTH after excision of affected parathyroid glands in normal locations, cervical thymectomy was performed. Ex vivo counts were used to confirm successful parathyroid excision with specimen radioactivity of >20% of the background count.^21^ When an ectopic intrathyroidal parathyroid adenoma is suspected, the ipsilateral thyroid is examined and scanned with a gamma probe for areas of increased radionuclide counts. Thyroidotomy is then performed over the region with the highest gamma counts using an electrocautery. The parathyroid adenoma was enucleated, with excision confirmed by ex vivo counts >20% above the background level. This technique facilitates the enucleation of the abnormal parathyroid gland, preventing thyroid lobectomy and preserving healthy thyroid tissue.^22^

One unresolved challenge for parathyroid surgeons is determining when to perform a bilateral neck exploration during MIP. Most surgeons use IoPTH to determine the extent of exploration and aim to achieve at least a 50% reduction in IoPTH levels to confirm intraoperative cure. However, the testing turnaround time for IoPTH can vary from 20 to more than 40 minutes. If the IoPTH levels do not drop appropriately after waiting for more than 20 minutes, surgeons will continue exploration, which can significantly extend the duration of the operation. Our study explored an expanded use of radioguidance to differentiate between SGD and MGD, based on the RR of excised parathyroid tissue. When the RR of the resected parathyroid gland is ≥1.2, there is an 85.3% chance of patient having an SGD, and surgeons can hold off on bilateral neck exploration to wait for IoPTH. Tobin et al. also attempted to use ex vivo radioactivity to identify MGD in primary HPT.^23^ They discovered that 69.4% of MGD patients had ex vivo counts below 50% of background counts. The PPV and NPV for 50% cutoff to predict MGD were 42.1% and 89.9%, respectively. They also found that a 100% cutoff had a PPV of 93.2% for SGD. Their cutoff value was very close to the cutoff value in this study. However, in their study, establishing 1.0 as the cutoff value for RR seemed arbitrary and lacked evidence support. Other studies attempted to use preoperative laboratory and imaging results to predict SGD versus MGD in primary HPT. Kekebew et al. used a dichotomous scoring model with 5 variables, including preoperative serum calcium ≥12 mg/dL, intact PTH ≥times the upper limit of normal PTH levels, sestamibi scan positive for SGD, neck US positive for SGD, and concordant sestamibi and neck US result.^24^ A total score of 3 or greater had a sensitivity of 43.9% and a specificity of 100% for correctly predicting single-gland primary HPT. The study stated that 35% of study cohort had a total score of 3 or higher. While this study showed promising results, one limitation of using this scoring system is that not all practices routinely obtain preoperative sestamibi and neck US. Additionally, with the growing recognition of primary HPT, there is an increasing number of patients with slightly elevated levels of calcium and PTH at the time of diagnosis. Consequently, fewer patients will have a total score of 3 or higher, making this scoring system less applicable. Mazeh et al. analyzed 1235 patients who underwent parathyroidectomy and developed a “Wisconsin Index” Nomogram to predict the likelihood of MGD after the resection of the first parathyroid gland. They defined the Wisconsin Index (WIN) as the preoperative serum calcium multiplied by the preoperative PTH and categorized WIN into 3 groups: low (<800), medium (801-1600), and high (>1600). The nomogram uses the weight of the removed parathyroid gland and the patient’s WIN category to estimate the likelihood of MGD. It does not take into account preoperative imaging, making it more suitable for general practice. Sepadari et al. developed a composite MGD score that integrates the 4D CT MGD score, which considers both the number and size of lesions on CT, along with WIN. The total score ranges from 0 to 6.^6^ Scores of ≥4, ≥5, and 6 demonstrated specificities of 81%, 93%, and 98%, respectively. These findings were further validated in their subsequent prospective study.^7^ Bunch et al. proposed using the second-largest high-confidence candidate lesions on 4D CT scan to predict MGD and the need for gland removal.^25^ They found sensitivities of 53% and 67%, and specificities of 96% and 96% for MGD when the second-largest lesion had a volume ≥60 mm^3^ and maximum diameter ≥7mm, respectively. For gland removal, when the second-largest lesion had a volume ≥114 mm^3^, sensitivity and specificity were 84% and 97%, and for maximum diameter ≥7mm, they were 93% and 84%, respectively.

This study has several limitations, including those inherent to any retrospective studies. The threshold of RR ≥1.2 was intended to predict the likelihood of SGD versus MGD, but it does not predict an intraoperative cure. We still need to rely on the IoPTH monitoring. Nonetheless, using this simple RR threshold can help surgeons make quick intraoperative decisions and reducing OR time. Our current practice is to begin by exploring the superior and inferior parathyroid glands on the same side. If an abnormal parathyroid gland is found, we remove it. If the resected parathyroid gland has an RR ≥1.2, we will wait for IoPTH result to confirm intraoperative cure or explore the contralateral side. If RR is less than 1.2, we will send the IoPTH and start exploring the other side simultaneously while waiting for the IoPTH result to shorten the unnecessary wait time. Another limitation of this study is the inevitable selection bias resulting from its single-institutional nature. The predictive values of the study are affected by the incidence of SGD among the study cohorts. According to the American Association of Endocrine Surgeons guidelines, the overall prevalence of SGD is 67%-94%.^4^ In this study, the frequency of SGD was low, likely because of the referral pattern at a tertiary medical center.^26^ The PPV of using RR ≥1.2 as the threshold is expected to be different (likely higher) in a different patient population. Although our study results were validated on a different cohort of 115 patients, the external validity is best confirmed by a prospective multi-institutional study. Additionally, we excluded patients with persistent HPT following parathyroidectomy, which could introduce further selection bias. Lastly, radioguided parathyroidectomy is not a standard practice in all institutions, and purchasing equipment such as probes and monitors will incur additional costs. However, the initial investment will be quickly recouped through reduced unnecessary OR wait time.

## Conclusions

Ex vivo radioactivity measurement provides an immediate and reliable prediction of parathyroid pathology. This simple intraoperative measurement could be used as an adjunct to guide surgical decision-making to distinguish SGD and MGD during parathyroidectomy in a time-efficient manner.

## Data Availability

The data underlying this article will be shared on reasonable request to the corresponding author.
